# Supplementary material for: Temporal discounting correlates with directed exploration but not with random exploration
Source: Sci Rep. 2020 Mar 4;10:4020. doi: 10.1038/s41598-020-60576-4 (PMC7055215; doi:10.1038/s41598-020-60576-4)
Supplement: Supplementary file 1 — Supplementary Information. [file 41598_2020_60576_MOESM1_ESM.pdf]

Supplementary Materials
"Temporal discounting correlates with directed exploration but not with random exploration"

Hashem Sadeghiyeh[1,3,*], Siyu Wang[1], Maxwell R. Alberhasky[4], Hannah M. Kyllo[1], Amitai Shenhav[5], and Robert C. Wilson[1,2]

[1]Department of Psychology, University of Arizona
[2]Cognitive Science Program, University of Arizona
[3]Department of Psychological Science, Missouri University of Science and Technology
[4]McCombs School of Business, University of Texas at Austin
[5]Department of Cognitive, Linguistic, & Psychological Sciences, Brown University
[*]Correspondence to: sadeghiyeh@email.arizona.edu

Supplementary Materials

1- Model fitting

In addition to model-free parameters (p(high info) and p(low mean)), We used a simple logistic model to fit the decisions on the first free trial (trial 5). We hypothesized that the value of each option (a or b) is dependent on 3 main parameters: R, the average reward of the option (based on previous trials); I, the information, defined as whether choosing the option would provide you with valuable information so the less information we have about an option the more its information bonus; and s, spatial location, we hypothesized some subjects might be biased in choosing right over left option or vice versa. Therefore, we can express the value of each option as:

$$Q_a = R_a + \alpha I_a + Bs_a \tag{1}$$

where α is called information bonus and B is the spatial bias.

We further assume the value of each option is perturbed with a logistic noise with the standard deviation of σ_d. So the probability of choosing option a over b, assuming the participants have a linear utility function, will be calculated as:

$$p_a = \frac{1}{1 + exp(\frac{Q_b - Q_a}{\sigma_d})} \tag{2}$$

Replacing Q_a and Q_b with their equivalent from equation (2):

$$p_a = \frac{1}{1 + exp(\frac{R_b - R_a + \alpha(I_b - I_a) + B(s_b - s_a)}{\sigma_d})} \tag{3}$$

We simply define the information (I) in a way that when option b is more informative than a, $I_b - I_a = +1$ and if option a is more informative than b, $I_b - I_a = -1$ and if they are equal in information $I_b - I_a = 0$. Similarly, the location variable is defined in a way that $s_b - s_a = +1$ if option b is on the right and $s_b - s_a = -1$ if it is on the left.

By fitting this model into our data, we are able to obtain information bonus (α), spatial bias (B) and decision noise (σ_d) for each participant. We expect that information bonus (α) would be highly correlated with our model-free parameter p(high info) and decision noise (σ_d) with p(low mean).

2- Model-based Results

Figure S1 shows the scatter plots between corresponding model-free and model-based parameters. As it was expected, we see a high correlation between information bonus and p(high info) and between decision noise and p(low mean) in both horizon. Model-based directed exploration (as defined by: information bonus h6 - information bonus h1) and model-based random exploration (decision noise h6 - decision noise h1) were also highly correlated with model-free directed and random explorations, respectively. Figure S2 shows the scatter plots and correlations between temporal discounting and the model-based parameters. The relationships are almost the same as the relations between temporal discounting and model-free parameters. Given this high correspondence, we based our main analyses in the manuscript on the model-free parameters.

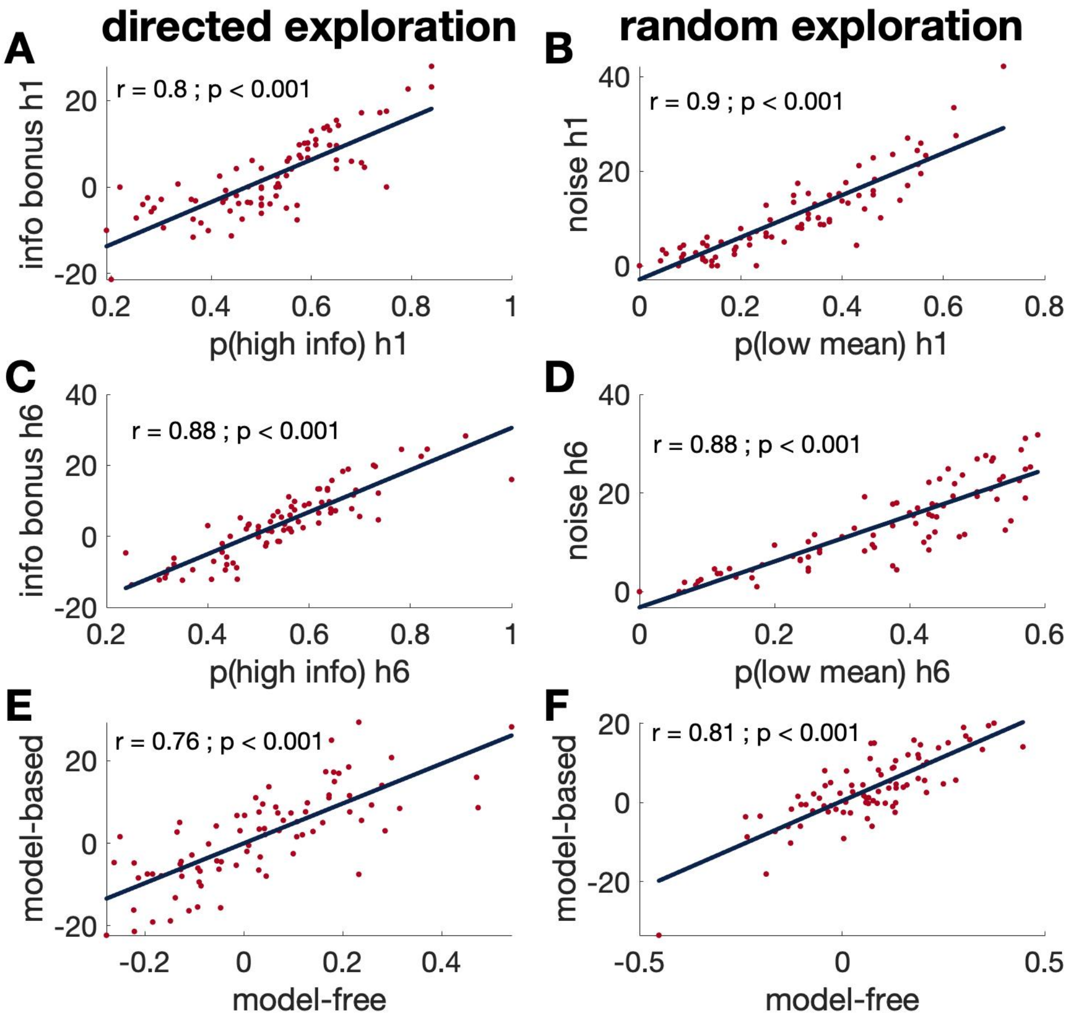

Figure S1: Scatterplots / Correlations between model-free and model-based parameters: (A) information bonus (from model) and p(high info) in horizon 1, (C) information bonus and p(high info) in horizon 6, (E) model-based directed exploration = information bonus h6 - information bonus h1, model-free directed exploration = p(high info) h6 - p(high info) h1, (B) decision noise (from model) and p(low mean) in horizon 1, (D) decision noise and p(low mean) in horizon 6, (F) model-based random exploration = decision noise h6 - decision noise h1, model-free random exploration = p(low mean) h6 - p(low mean) h1

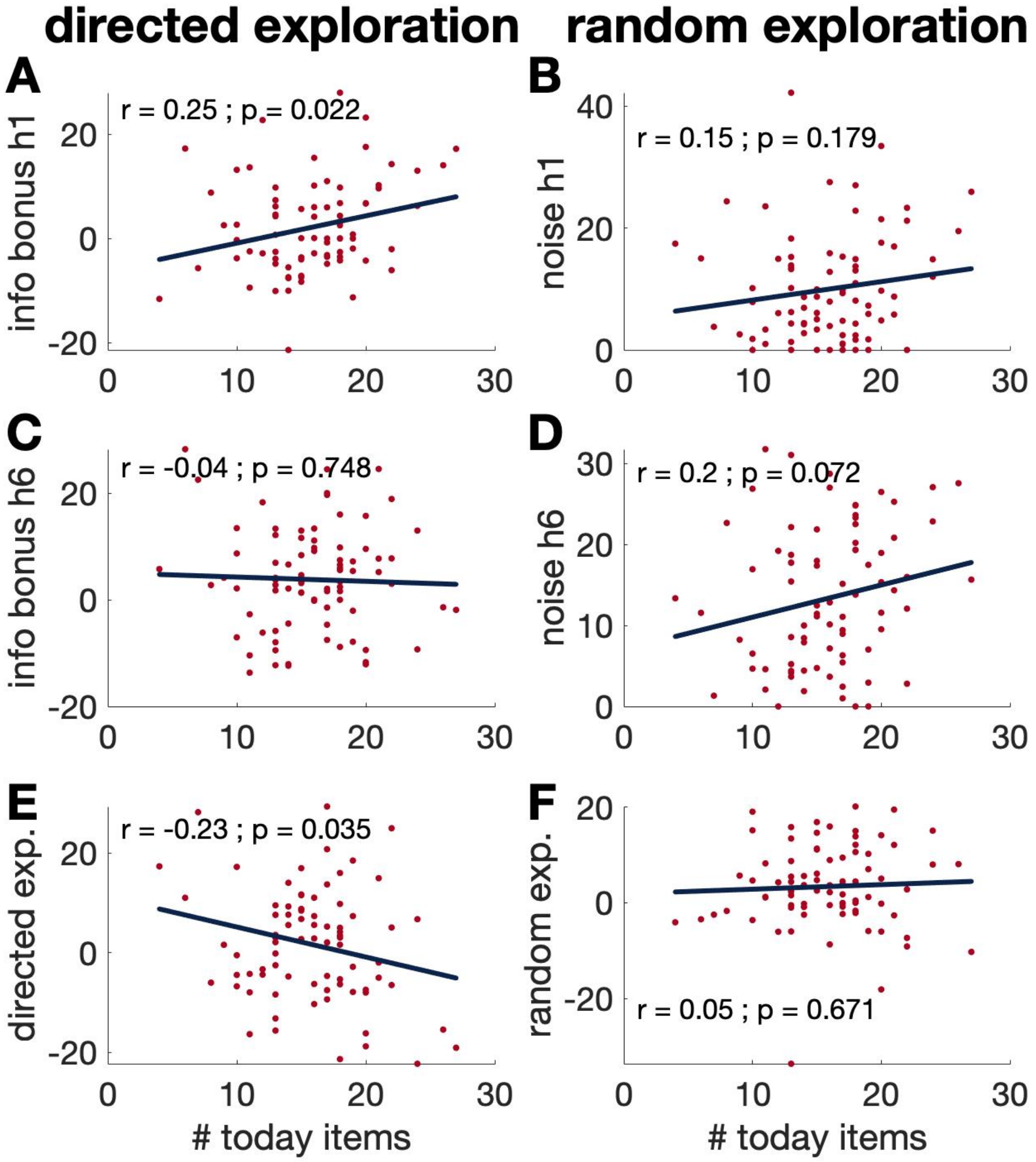

Figure S2: Scatter plots / correlations for model-based parameters over a temporal discounting measure (# today items). (A) information bonus at horizon 1, (B) decision noise at horizon 1, (C) information bonus at horizon 1, (D) information bonus at horizon 6, (E) model-based directed exploration (= info bonus h6 - info bonus h1, and (F) model-based random exploration (= noise h6 - noise h1. It yields the exact same conclusion we reached by a model-free analysis

3- Correlations between temporal discounting measures

Figure S3 shows that there is high correlations between different measures of temporal discounting in our study. More specifically, there is high correlations between a simple measure of temporal discounting, i.e. # today items: the total number of today (smaller immediate items) chosen by subject and more sophisticated measures (different k's) that utilize a model fitting (Pearson's correlation coefficients between .89 - 1). So we selected the simplest one (# today items) in our main analysis.

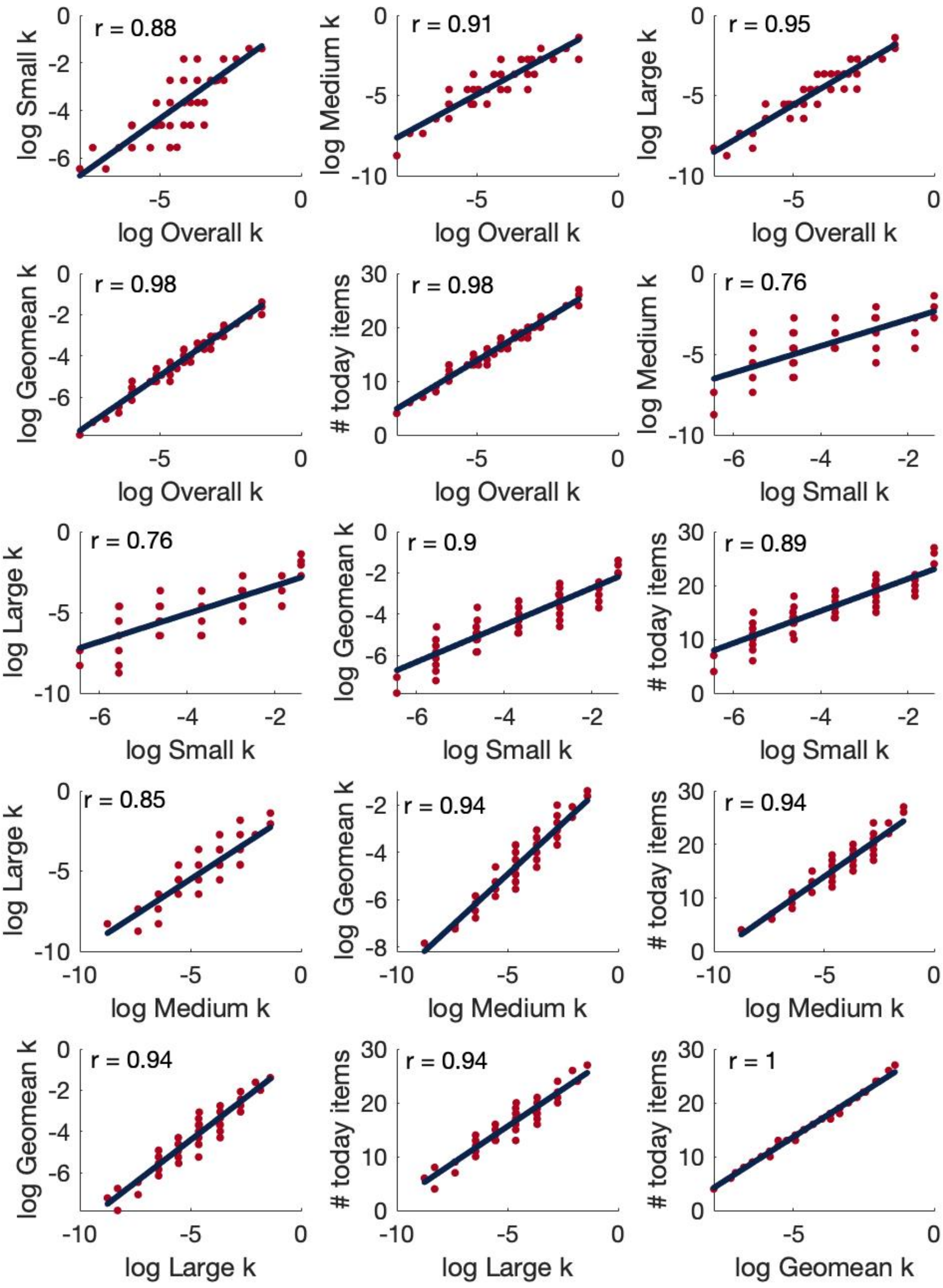

Figure S3: Scatterplots / Correlations between different measures of temporal discounting: log Overall k, log Small k, log Medium k, log Large k, log Geomean k, and # today items

4- Instructions for Horizon Task:

Page 1: 'Welcome! Thank you for volunteering for this experiment.'

Page 2: 'In this experiment you will do four things.

1. Baseline eye measurement. This will take about 5 minutes.
2. Play a gambling task in which you will make choices between two options. This will take about 20 minutes.
3. Play another gambling task which also takes about 20 minutes.
4. When you''re done with the tasks, there will be a short post-experiment survey.'

Page 3: 'Now we are going to get a baseline measurement of your eyes using the eye tracker. '

Page 4: 'To do this we need you to stare at the screen for 5 minutes. Feel free to relax and daydream, but please stay in the chin rest. Press space to continue'

Page 5: 'Press space to start the eye-measurement.'

After 5 minutes baseline eye tracker:

Page 1: 'Welcome! Thank you for volunteering for this experiment.'

Page 2: 'In this experiment - the gambling task - we would like you to choose between two one-armed bandits of the sort you might find in a casino.'

Page 3: 'The one-armed bandits will be represented like this'

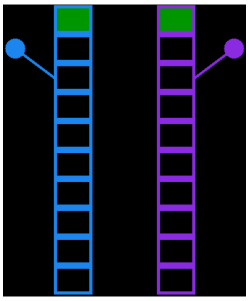

Page 4: 'Every time you choose to play a particular bandit, the lever will be pulled like this ...'

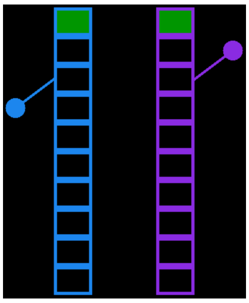

Page 5: '... and the payoff will be shown like this. For example, in this case, the left bandit has been played and is paying out 77 points.'

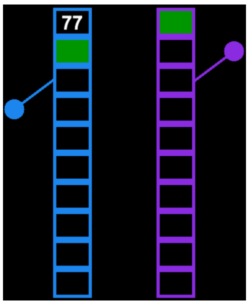

Page 6: 'During one game, each bandit tends to pay out about the same amount of reward on average, but there is variability in the reward on any given play.'

Page 7: 'For example, the average reward for the bandit on the right might be 50 points, but on the first play we might see a reward of 52 points because of the variability ...'

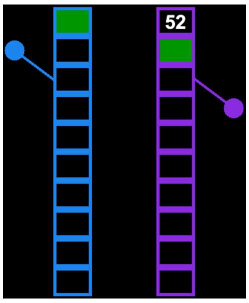

Page 8: '... on the second play we might get 56 points ... '

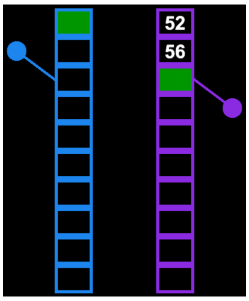

Page 9: '... if we open a third box on the right we might get 45 points this time ... '

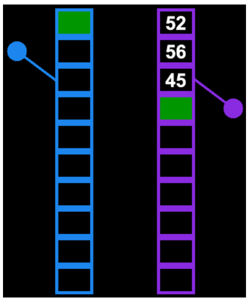

Page 10: '... and so on, such that if we were to play the right bandit 10 times in a row we might see these rewards ...'

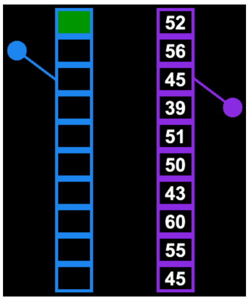

Page 11: 'Both bandits will have the same kind of variability and this variability will stay constant throughout the experiment.'

Page 12: 'During one game, one of the bandits will always have a higher average reward and hence is the better option to choose on average.'

Page 13: 'To make your choice: Press <- to play the left bandit Press -> to play the right bandit'

Page 14: 'On any trial you can only play one bandit and the number of trials in each game is determined by the height of the bandits. For example, when the bandits are 10 boxes high, there are 10 trials in each game ... '

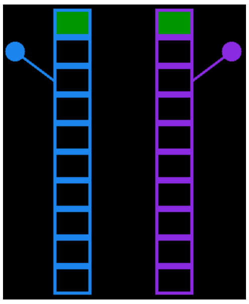

Page 15: '... when the stacks are 5 boxes high there are only 5 trials in the game.'

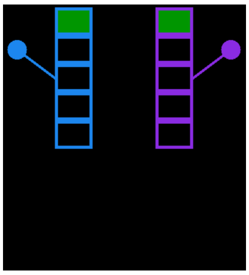

Page 16: 'In addition, the first 4 choices in each game are instructed trials where we will choose an option for you. This will give you some experience with each option before you make your first choice.'

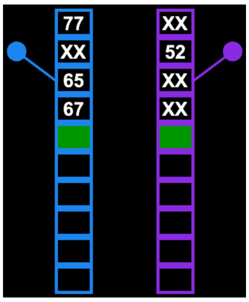

Page 17: 'These instructed trials will be indicated by a green square inside the box we want you to open and you must press the button to choose this option in order to move on to see the reward and move on the next trial. For example, if you are instructed to choose the left box on the first trial, you will see this:'

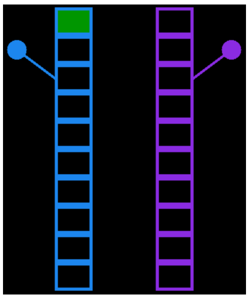

Page 18: 'If you are instructed to choose the right box on the second trial, you will see this:'

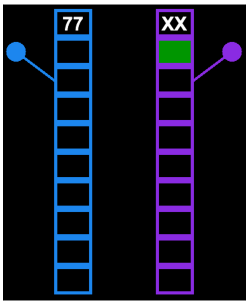

Page 19: 'Once these instructed trials are complete there will be a go-cue and a beep, and then you will have a free choice between the two stacks that is indicated by two green squares inside the two boxes you are choosing between.'

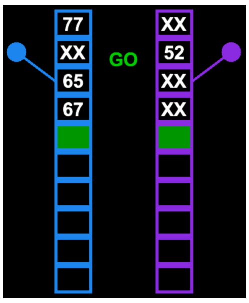

Page 20: 'Throughout the task we will be tracking your eyes. To help us better track your eyes, the timing of the task is quite slow. Each game begins with the presentation of a fixation cross like this ... please try to stare at this cross while it is displayed.'

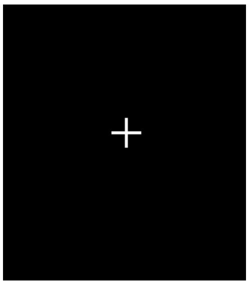

Page 21: 'Then, the fixation cross will disappear and the bandits will appear - like this ... during this period feel free to look where you want to.'

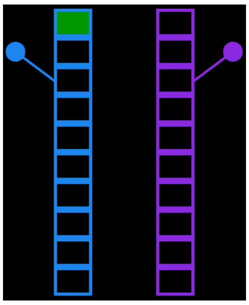

Page 22: 'Then, a GO cue will appear at which point you need to choose between the two options'

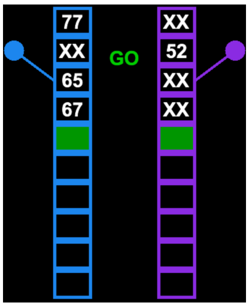

Page 23: 'So ... to be sure that everything makes sense let's work through an example game ... Press <- to play the left bandit Press -> to play the right bandit'

Page 25: 'Good job! Now you know how to play this game.'

Page 26: 'Press space when you are ready to begin. Earn as many points as you can! Good luck! Remember to stay in the chin rest!'
